# Duality of Interactions Between TGF-β and TNF-α During Tumor Formation

**DOI:** 10.3389/fimmu.2021.810286

**Published:** 2022-01-05

**Authors:** Zhi-wei Liu, Yi-ming Zhang, Li-ying Zhang, Ting Zhou, Yang-yang Li, Gu-cheng Zhou, Zhi-ming Miao, Ming Shang, Jin-peng He, Nan- Ding, Yong-qi Liu

**Affiliations:** ^1^ Provincial-Level Key Laboratory for Molecular Medicine of Major Diseases and The Prevention and Treatment with Traditional Chinese Medicine Research in Gansu Colleges and Universities, Gansu University of Chinese Medicine, Lanzhou, China; ^2^ Gansu Institute of Cardiovascular Diseases, The First People’s Hospital of Lanzhou City, Lanzhou, China; ^3^ Key Laboratory of Space Radiobiology of Gansu Province & Key Laboratory of Heavy Ion Radiation Biology and Medicine of Chinese Academy of Sciences Institute of Modern Physics, Chinese Academy of Sciences, Lanzhou, China; ^4^ Key Laboratory of Dunhuang Medicine and Transformation at Provincial and Ministerial Level, Gansu University of Chinese Medicine, Lanzhou, China

**Keywords:** TGF-β, TNF-α, proliferation, apoptosis, inflammation, genomic instability, epithelial-mesenchymal transition

## Abstract

The tumor microenvironment is essential for the formation and development of tumors. Cytokines in the microenvironment may affect the growth, metastasis and prognosis of tumors, and play different roles in different stages of tumors, of which transforming growth factor β (TGF-β) and tumor necrosis factor α (TNF-α) are critical. The two have synergistic and antagonistic effect on tumor regulation. The inhibition of TGF-β can promote the formation rate of tumor, while TGF-β can promote the malignancy of tumor. TNF-α was initially determined to be a natural immune serum mediator that can induce tumor hemorrhagic necrosis, it has a wide range of biological activities and can be used clinically as a target to immune diseases as well as tumors. However, there are few reports on the interaction between the two in the tumor microenvironment. This paper combs the biological effect of the two in different aspects of different tumors. We summarized the changes and clinical medication rules of the two in different tissue cells, hoping to provide a new idea for the clinical application of the two cytokines.

## Introduction

The process of tumor formation is complex and changeable and closely related to changes in its microenvironment. As an important part of the tumor microenvironment, cytokines have a two-way relationship with cancer. On the one hand, cytokines can directly affect carcinogenesis and metastasis by changing the phenotype of tumors. On the other hand, cytokines can also play a role through host immune system to produce specific responses against tumors (1). The two most mentioned cytokines in the tumor microenvironment are TGF-β and TNF-α. Interestingly, these two cytokines play a two-way role in the process of tumor formation, that is they can both inhibit tumor formation and promote tumor development (2). However, the interaction between TGF-β and TNF-α during tumor formation is still unclear. This article reviews the two sides of the interaction between TGF-β and TNF-α during tumor formation.

## Role of TGF-β in the Process of Tumor Formation

TGF-β was originally thought to induce the proliferation of rat renal fibroblasts (3). In normal environment, TGF-β, as an anti-inflammatory factor, has been proved to inhibit the production and function of effector T cells and antigen-presenting dendritic cells, and can regulate natural killer cells, macrophages, dendritic cells and granulocytes to inhibit inflammation (4, 5). Interestingly, in tumors, its effects are multifaceted, depending on the stage of the tumor. In the early stage, TGF-β, as an effective growth inhibitor, can inhibit the epithelial cell cycle and promote cell apoptosis, thus inhibiting the occurrence and development of tumors (6). In the middle and late stages, TGF-β can induce and promote epithelial-mesenchymal transformation (EMT), increase the activity and invasiveness of tumor cells and participate in tumor malignant progression and angiogenesis (7). In view of the above phenomenon, some literatures have proposed explanations. In the early stage, TGF-β is involved in the inhibition and apoptosis of tumor cells as a major tumor suppressor factor. In the middle and late stages, tumor cells develop resistance to TGF-β or are reinterpreted by tumor cells to promote tumor growth (8). Therefore, TGF-β plays an important role in tumor formation and development.

## Role of TNF-α in the Process of Tumor Formation

Tumor necrosis factor (TNF) family is an important class of cytokines, which play an important role in the regulation of a series of physiological and pathological reactions such as cell proliferation, differentiation, apoptosis, immune responses and inflammation (9). TNF-α is produced by monocytes and macrophages, it can not only regulate the immune function and cause necrosis of some tumor cells, but also mediate pathophysiological reactions such as inflammatory processes, tissue injury and shock (10). The proinflammatory effect of TNF-α can promote the formation of tumors. Studies have shown that TNF-α can lead to highly invasive diseases in many malignant tumors, and effectively increase the transcriptional levels of different inflammatory factors and chemokines, and also increase the metastatic phenotype of cancer cells to promote the progression of cancer, so it plays an important role in regulating the proliferation, migration and invasion of various types of cancer cells (11–13). The bidirectional regulatory effect of TNF-α on tumors also plays a key role in cancer progression.

## Interaction Between TNF-α and TGF-β

TNF-α is recognized as a strong pro-inflammatory factor and an important mediator of immune protection. But the role of TGF-β is complicated. Although it has a pro-inflammatory effect, it is more regarded as an effective immunosuppressive cytokine. Both of them are important cytokines involved in the process of tumor formation, and their interaction relationship mainly depends on the influence of the microenvironment.

TNF-α and TGF-β can promote each other’s production, and TNF-α has been shown to affect TGF-β expression in many cells and tissues, such as endothelial cells, lung epithelial cells, macrophages, as well as subcutaneous adipose tissue (14). A correlation was also found between TNF-α mRNA expression and TGF-β mRNA expression in fibroblasts as well as in thyroid cells (15–17). Similarly, TGF-β can also activate the expression of TNF-α in *in vitro* and *in vivo* experiments and plays an important role in tissue injury repair, inflammation and tumor growth (18–21). In addition to direct mutual stimulation of the two, TNF-α has an effect on TGF-β receptors, and TNF-α can increase TGF-β type I and type II receptor expression and stimulate Smad3 phosphorylation in rat fibroblasts (22). The mutual stimulation of the two is also closely related to their respective signaling pathways, and TNF-α can lead to increased TGF-β1 mRNA and promote the expression of TGF-β1 by activating the extracellular regulated kinase (ERK) specific pathway in fibroblasts (23). Subsequent in-depth studies revealed that TNF-α can induce the expression of activator protein 1 (AP-1) and allow it to bind to DNA, which leads to increased transcription of the TGF-β1 gene (24). There are also relevant reports pointing out that up-regulation of TGF-β1 expression induced by TNF-α may be associated with activation of the NF-κB pathway (25–27). TGF-β1 and TNF-α have mutually stimulating effect, and their mutual induction and crosstalk in their respective pathways leads to non-destructive tissue remodeling, ultimately leading to myocardial fibrosis, dysfunction and heart failure (28).

TNF-α and TGF-β can also inhibit each other, and increased TNF-α can inhibit TGF-β-mediated gene or signaling pathway conduction in the previous literature (29–31). It has also been shown that elevated TGF-β content can also inhibit the expression of TNF-α and its receptor in different cell lines, TGF-β can be produced by autocrine means in microglia, and can inhibit TNF-α production and prevent oxidative stress response, a phenomenon that contributes to the survival of phagocytic microglia (32). TGF-β inhibited the induction of TNF-α expression at both protein and mRNA levels in rat astrocytes (33). In phagocytes, TGF-β inhibited TNF-α production by mediating the expression of the major antigen and adhesion-promoting protein (BAD1) in type B dermatitis (34). In human venous endothelial cells (EC), TGF-β can down-regulate TNF-α receptors, thus exerting an immunosuppressive effect (35). TNF-α and TGF-β can interact differently in different microenvironments of different cell lines, which may be the result of self-regulation by the body in order to maintain the homeostasis of the normal microenvironment. However, the mechanism of the interaction between the two on tumorigenesis is still unclear. There are ten hallmarks which have been published about the process of normal cells that transforming into tumor cells, including self-sufficient growth signals, insensitivity to growth signals, avoidance of apoptosis, limit potential replication, sustained angiogenesis, tissue invasion and metastasis, avoidance of immune destruction, promotion of tumor inflammation, deregulating cellular energetics and genome instability and mutation (36). However, it has been investigated that the interaction between TNF-α and TGF-β is also two-sided during tumor formation. The authors reviewed the dual roles of proliferation, apoptosis, inflammatory response, immune regulation, EMT, tissue invasion and metastasis, and genomic instability in the process of tumor formation.

### Role of TNF-α and TGF-β in Cell Proliferation

The first step in the transformation of normal cells into cancer is often abnormal changes in proliferation ability, and TNF-α and TGF-β play multiple roles in the regulation of cell proliferation, as shown in **Table 1**. First of all the two have a two-way effect in the coordinated regulation of proliferation, which can synergistically enhance the proliferation ability of tumor cells and increase the characteristics of tumor stem cell in pancreatic cancer cells (MiaPaCa-2) (37). In rheumatoid synovial fibroblasts (RSF), TNF-α and TGF-β can synergistically stimulate the proliferation of RSF, which is related to the activated RAS gene (38). It is found in myofibroblasts (MFBIC) that although TGF-β has a slight inhibitory effect on the cell growth, when used in combination with TNF-α, TGF-β can promote cell proliferation and stimulate the formation of liver fibrosis (39). In addition to the synergistic promotion, there is also a synergistic inhibitory effect. In human promyelocytic leukemia cells (HL-60), both inhibit cell growth, which is associated with down-regulation of c-myc expression (40). In hematopoietic stem cells, hematopoietic progenitor cells and cord blood megakaryocytes (MK), TNF-α and TGF-β can inhibit cell proliferation and colony-forming ability (41, 42). It has also been found that in mouse hepatocytes, TGF-β1 can inhibit the production of hepatic growth factor (HGF), while TNF-α is positively correlated with the production of TGF-β, and inhibiting the level of TNF-α can inhibit the production of TGF-β1, thereby enhancing liver tissue regeneration (43). TNF-α and TGF-β also have antagonistic effect on proliferation. In human fibroblasts and nasal epithelial cells (HNECs), TGF-β dose-dependently inhibits TNF-α-stimulated cell proliferation (44, 45). In the normal central nervous system, increased TGF-β1 inhibits the proliferation of brain endothelial cells (BEC), whereas in cerebral ischemia or other neurotic processes, activated microglia increase TNF-α production, which can promote BEC proliferation (46).

**Table 1:** The mechanism of TNF-α and TGF-β on proliferation in different cells.

Tissue/cell	Mechanism
**Synergy effect**	
**MiaPaCa-2**	Enhance the proliferation ability and cancer stem cell characteristics of tumor cells (37)
**RSF**	Stimulation of RSF proliferation may be related to activated RAS genes (38)
**MFBIC**	They can promote the proliferation of cells and stimulate the formation of liver fibrosis (39)
**HL-60**	Inhibition of cell growth may be associated with down-regulation of c-myc expression (40)
**Hematopoietic stem cells, etc.**	Inhibition of cell proliferation and colony formation (41, 42)
**Mouse hepatocytes**	Inhibition of the level of TNF-α can inhibit the production of TGF-β1, thereby enhancing liver tissue regeneration (43)
**Antagonism effect**	
**Human fibroblasts and nasal epithelial cells**	TGF-β dose-dependently inhibited the cell proliferation ability stimulated by TNF-α (44, 45)
**BEC**	TGF-β1 inhibits the proliferation of brain endothelial cells, but TNF-α promotes BEC proliferation (46)

In summary, in pancreatic cancer, rheumatoid synovial fibroblasts and myofibroblasts, TNF-α and TGF-β can promote cell proliferation, while in human promyelocytic leukemia cells and hematopoietic cells, the two can inhibit cell proliferation. In fibroblasts, nasal epithelial cell and neurological diseases, TGF-β can restrain the pro-proliferative effect of TNF-α.

### Role of TNF-α and TGF-β in Apoptosis

**Figure 1:**
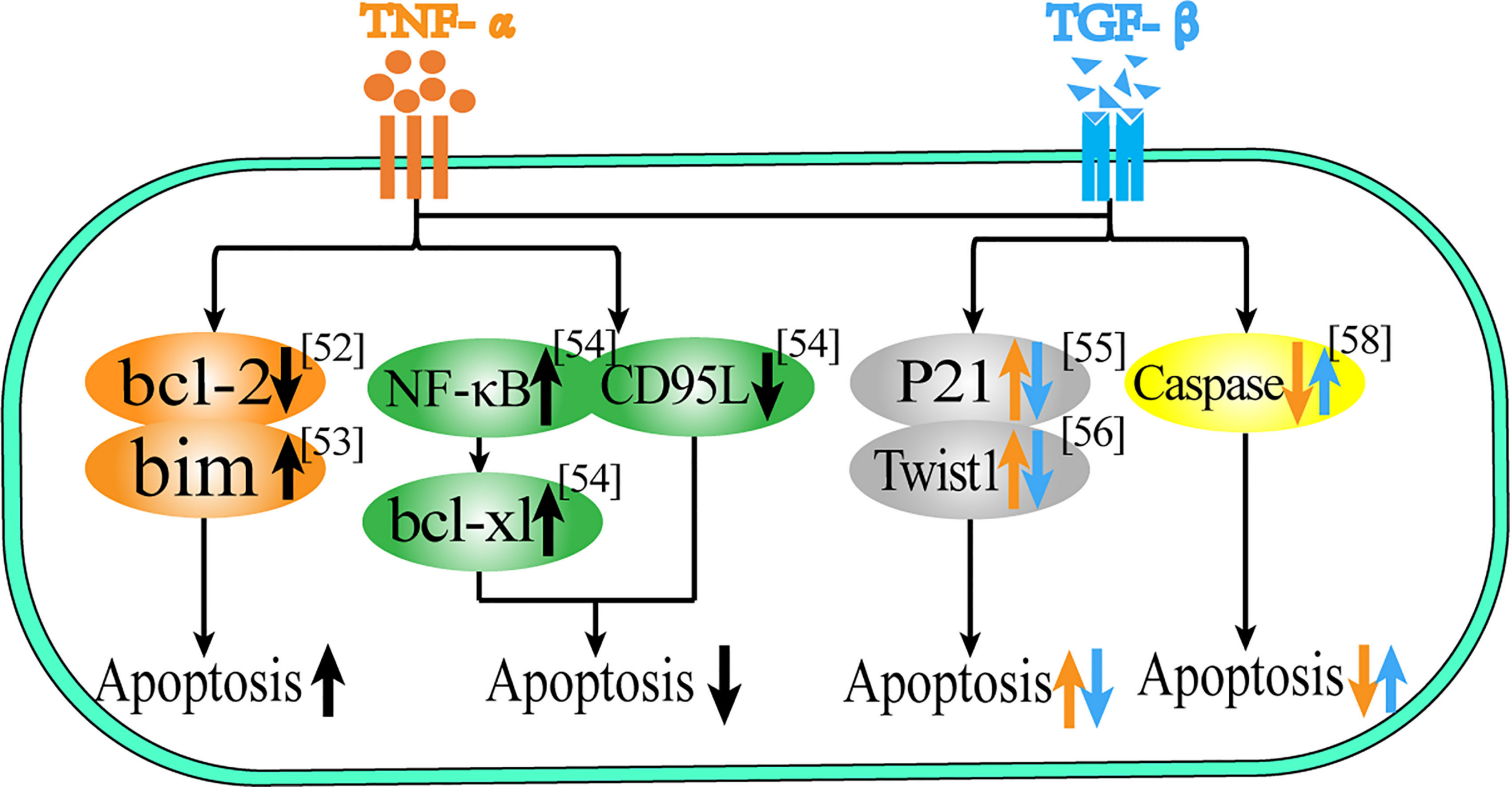
Role of TNF-α and TGF-β in apoptosis. This figure summarizes the regulatory effect of TNF-α and TGF-β on different proteins in terms of apoptosis. The black arrow represents the common regulatory role of TNF-α and TGF-β, and the orange arrow represents the effect of TNF-α, the blue arrow represents the effect of TGF-β.

In addition to abnormal proliferation, avoiding cell apoptosis is also an important manifestation in the process of cell carcinogenesis, as shown in **Table 2** and **Figure 1**. For Schwann cells (SC), oligodendrocytes (OLG), human umbilical vein endothelial cells (HUVEC) and malignant glioma cells (SMA-560), both cytokines have a significant synergistic effect on promoting apoptosis (54–58), and they can also induce the expression of apoptosis-related proteins and play a synergistic effect. In HL-60 cells, TGF-β1 and TNF-α can induce apoptosis by down-regulating the expression of the anti-apoptotic protein Bcl-2 (47). In gastric cancer cells (SNU620), both synergistically promote up-regulation of the transcription level of the pro-apoptotic protein Bim, thereby promoting apoptosis (48). However, in hepatic stellate cells (HSCs), TGF-β and TNF-α can synergistically inhibit apoptosis levels not only by reducing the expression of the spontaneously apoptotic CD95L gene but also by inducing NF-κB activation and up-regulating the anti-apoptotic protein Bcl-xL (49).

**Table 2:** The mechanism of TNF-α and TGF-β on apoptosis in different cells.

Tissue/cell	**Mechanism**
**Synergy**	
**HL-60 cells**	Apoptosis was induced by down regulating the expression of Bcl-2 (47)
**SNU620**	The up regulation of Bim transcription may be related to the activation of JNK and Smad3 signaling pathways (48)
**HSC**	Down regulation of CD95L gene expression and activation of NF-κB and up regulation of Bcl-xL (49)
**Antagonism**	
**Epithelial Cells**	TGF-β1 induces apoptosis in epithelial cells, while TNF-α regulates the level of apoptosis through P21 (50)
**Melanoma cells**	The opposite phenomenon in the number of cell death may be related to the regulation of Twist1 protein level (51)
**Splenocyte**	TGF-β is anti-apoptotic, while TNF-α exacerbates apoptosis (52)
**MC3T3-E1**	TGF-β1 attenuates TNF-α-induced caspase gene expression to reduce TNF-α-induced apoptosis of rat osteoblasts (53)

In terms of apoptosis, there is not only a synergistic effect but also a mutual antagonism between the two. In epithelial cells, TGF-β1 can induce epithelial cell apoptosis, while TNF-α can induce the anti-apoptotic protein P21 to regulate the level of apoptosis (50). In melanoma cells, TGF-β1 can induce cell death, while TNF-α can reduce the relative cell death number, a phenomenon that may be associated with both regulating Twist1 protein levels (51). In splenocytes, TGF-β has an anti-apoptotic effect, while TNF-α can exacerbate the apoptotic process (52). In osteoblasts (MC3T3-E1), TGF-β1 can attenuate TNF-α-induced caspase gene expression to reduce TNF-α-induced apoptosis in murine osteoblasts (53). Thus, TNF-α and TGF-β have multiple roles in regulating cell proliferation and apoptosis, and the release of these cytokines by the body indirectly determines the survival fate of cells.

Therefore, both of them can promote apoptosis in SC, OLG, HOVEC, SMA-560, HL-60 and gastric cancer cells, while in hepatic stellate cells, both of them can inhibit apoptosis. In epithelial cells and melanoma cells, TGF-β induces apoptosis while TNF-α is anti-apoptotic, and in splenocytes and osteoblasts, TGF-β is anti-apoptotic while TNF-α induces apoptosis.

### Role of TNF-α and TGF-β in Inflammation and Immunomodulation

**Figure 2:**
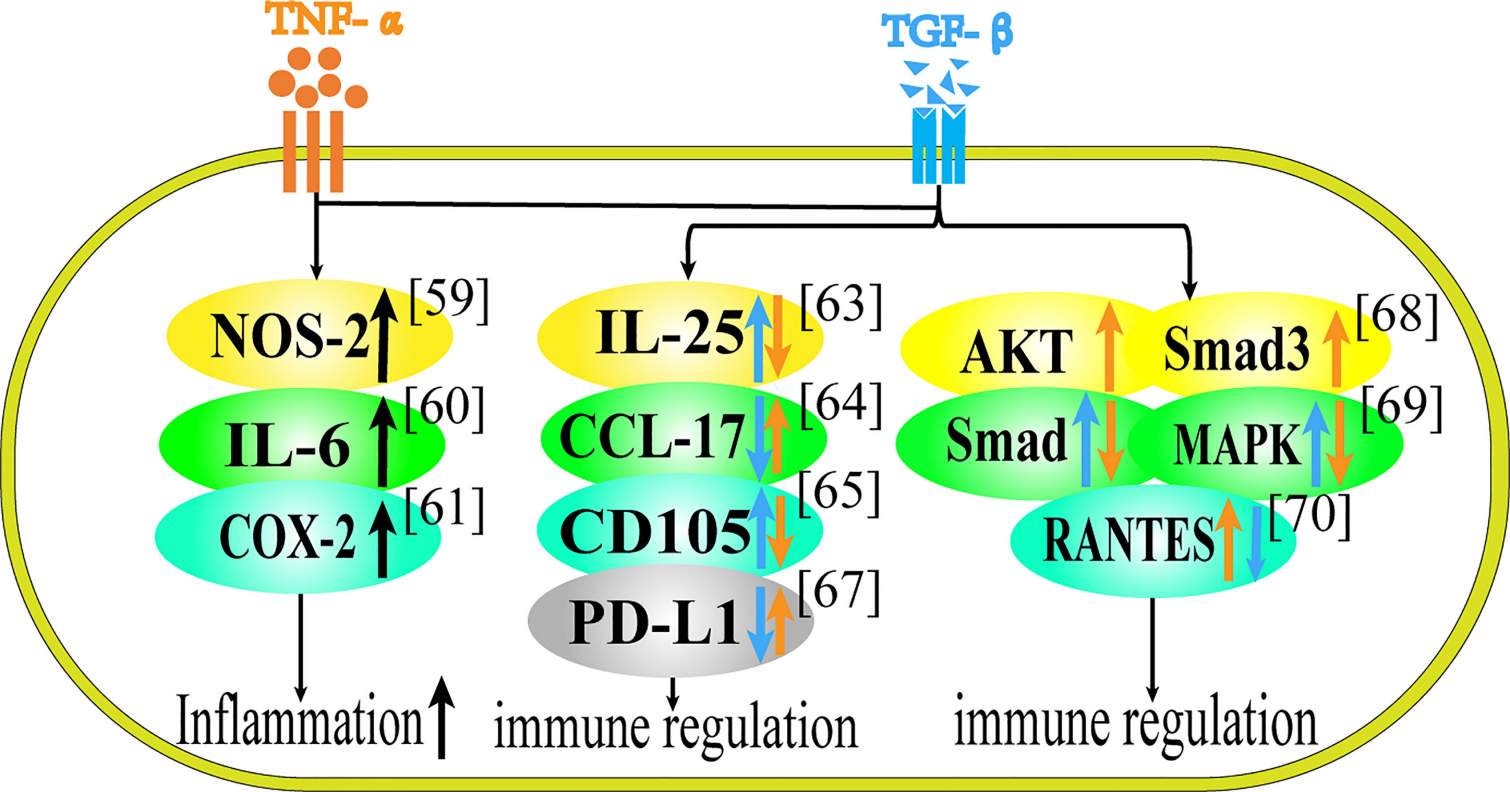
Role of TNF-α and TGF-β in Inflammation and Immune Regulation. This figure summarizes the regulatory effect of TNF-α and TGF-β on different proteins in terms of immune regulation. Black arrows represent the common regulatory effect of TNF-α and TGF-β. Orange arrows represent the effect of TNF-α, and blue arrows represent the effect of TGF-β.

Inflammation is an important intermediate process of carcinogenesis, and immune regulation disorders can induce the formation of cancer microenvironment. TNF-α and TGF-β are two important cytokines involved in immune regulation and inflammatory response. TNF-α and TGF-β also have two-way functions in immune regulation and participation in inflammation, as shown in **Table 3** and **Figure 2**. These functions may vary depending on the microenvironment.

**Table 3:** The mechanism of TNF-α and TGF-β on inflammation and immune regulation in different cells.

Tissue/cell	Mechanism
**Synergy**	
**Astrocytes**	Co-stimulation of TNF-α and TGF-β1 increased the expression of NOS-2 (59)
**Enterocyte**	Synergistically promotes IL-6 secretion (60)
**MSC**	Synergistically increase the release of proinflammatory factors such as COX-2 from MSCs (61)
**Antagonism**	
**Colon tissue**	TNF-α can inhibit the synthesis of IL-25, while TGF-β1 can stimulate the up-regulation of IL-25 in colon tissue (62)
**HaCaT**	TGF-β1 can inhibit TNF-α-induced CCL-17 production (63)
**vascular endothelial cell**	TNF-α down-regulated CD105 expression, whereas TGF-β1 up-regulated CD105 expression (64)
**Lupus cells**	TGF-β has the ability to suppress exogenous TNF-α to restore PD-L1 expression in lupus cells (65)
**Regulatory T cells**	TNF-α impairs TGF-β-induced Treg cell differentiation and function through Akt and Smad3 signaling (66)
**Fibroblasts**	TNF-α can inhibit TGF-β1-induced activation of Smad2/3 and p38 MAPK (67)
**Rheumatoid synovial fibroblasts**	Inhibition of TNF-α-induced RANTES expression by TGF-β1 may be associated with reduced NF-κB binding to the promoter (68)

The synergistic effect of TNF-α and TGF-β is mostly manifested in jointly promoting the release of inflammatory factors. In astrocytes, the co-stimulation of TNF-α and TGF-β1 increased the expression of NOS-2 compared with cytokine stimulation alone (59). TNF-α and TGF-β1 can synergistically increase IL-6 secretion in IEC-6 cells (60). In mesenchymal stem cells TNF-α and TGF-β can increase the release of proinflammatory factors such as COX-2, that is to say, in the presence of TNF-α, TGF-β1 is converted into pro-inflammatory cytokines, and when they act together on MSCs, they can play a synergistic effect in promoting inflammation (61). Besides, TGF-β can reverse the inhibitory effect of MSCs on T cell proliferation, and cooperate with TNF-α to promote immune response (69).

Through literature research, we found that the antagonistic effect of TNF-α and TGF-β have been reported in terms of immune regulation and inflammatory response. Regarding the antagonism of the two cytokines, studies have shown that in inflammatory bowel disease (IBD), TNF-α can inhibit the synthesis of IL-25, while TGF-β1 can stimulate the up-regulation of IL-25 in colon tissue (62). TGF-β1 can inhibit the production of CCL-17 in human epidermal cells induced by TNF-α, suggesting that TGF-β1 may have a certain effect on the treatment of atopic dermatitis (63). TNF-α can down-regulate the expression of CD105, while TGF-β1 can up-regulate it in vascular endothelial cells and this differential expression can regulate the damage repair of endothelial cells (64). TGF-β secreted by human umbilical cord mesenchymal stem cells can inhibit TNF-α and relieve atopic dermatitis (70). In patients with systemic lupus erythematosus (SLE), exogenous TNF-α can restore the expression of PD-L1 in lupus cells, while TGF-β, on the contrary, can inhibit the expression of PD-L1 in lupus cells (65).

Speaking of the antagonism of the two cytokines in their respective pathways, the increase of TNF-α can lead to the activation of AKT, and activated AKT can interact with Smad3, which leads to the inhibition of TGF-β pathway in regulatory T cells, so TNF-α can weaken the differentiation and function of Treg cells induced by TGF-β in autoimmune diseases through AKT and Smad3 signaling pathways (66). In fibroblasts, TNF-α can inhibit TGF-β1-induced activation of Smad2/3 and p38 MAPK pathway and terminate nerve growth factor (NGF) expression, which inhibits the regeneration of neurons (67). In rheumatoid synovial fibroblasts, TGF-β1 inhibits the expression of RANTES induced by TNF-α in a dose-dependent manner, possibly due to the decreased binding of NF-κB to the RANTES promoter (68).

In summary, TNF-α and TGF-β can increase the release of inflammatory factors and play pro-inflammatory or anti-inflammatory effect in different cells and tissues. In epidermal cells and patients with systemic lupus erythematosus, TNF-α is proinflammatory and TGF-β is anti-inflammatory. In vascular endothelial cells, inflammatory bowel disease, T cells and fibroblasts, TNF-α is anti-inflammatory and TGF-β is pro-inflammatory.

### Role of TNF-α and TGF-β in EMT and Tissue Invasion and Metastasis

**Figure 3:**
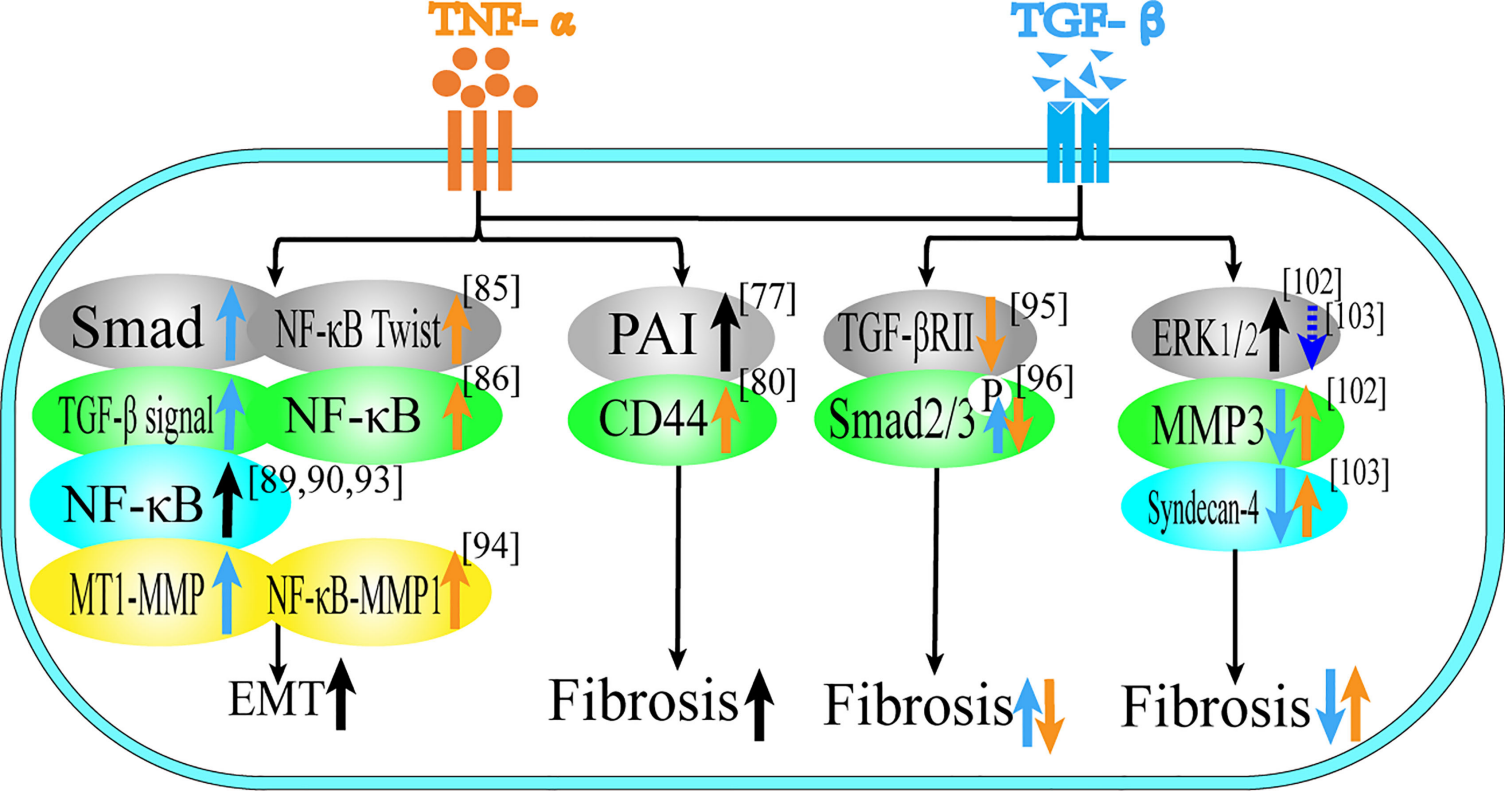
Role of TNF-α and TGF-β in EMT and tissue invasion and metastasis. This figure summarizes the regulatory effect of TNF-α and TGF-β on different proteins in terms of tissue invasion and metastasis, black arrows represent the common regulatory effect of TNF-α and TGF-β, reflecting the common regulatory effect of the two, orange arrows represent the effect of TNF-α, and blue arrows represent the effect of TGF-β.

EMT is an important process of cancer invasion and changes in cell-extracellular matrix (ECM) interactions also contribute to these pathological conditions (71–73). TNF-α and TGF-β play important roles in EMT and tumor formation, and they play different regulatory roles in different cell lines and different microenvironments, as shown in **Table 4** and **Figure 3**. In previous studies, a large number of literatures reported that the two play a synergistic role in fibrosis and EMT.

**Table 4:** The mechanism of TNF-α and TGF-β on EMT, tissue invasion and metastasis in different cells.

Tissue/cell	Mechanism
**Synergy**	
**kidney and lung tissues**	TNF-α and TGF-β cooperatively induce fibrosis through their respective pathways (74–76)
**retinal pigment epithelial cells**	TNF-α activates TGF-β signaling and induces the formation of fibrotic foci in retinal pigment epithelial cells (77)
**Bronchial epithelium**	Combined stimulation of bronchial epithelial cell transformation and cell migration (78)
**Breast cancer**	TGF-β and TNF-α induce EMT and form a stable breast cancer stem cell phenotype in breast cancer cells (79, 80)
**Cervical and ovarian cancer cells**	TNF-α and TGF-β synergistically induce cancer cell EMT through the NF-κB/Twist axis (81, 82)
**A549 cells**	TNF-α accelerates TGF-β1 to cause EMT in a Smad dependent manner (83)
**H460**	TNF-α and TGF-β can promote lung cancer stemness at H460 *via* NF-κB and FoxM1 pathways (84)
**3D cancer cells**	TNF-α and TGF-β1 can synergistically increase the migration rate and persistence of 3D cancer cells (85)
**Antagonism**	
**Fibroblasts**	TGF-β can stimulate the production of extracellular matrix of fibroblasts, while TNF-α has anti-fibrotic activity (86)
**Myofibroblasts**	TNF-α inhibits myofibroblast differentiation by inhibiting the phosphorylation of Smad2/3 by TGF-β1 (87)
**Fibroblasts**	TNF-α elevates the level of Smad7 and reduces the phosphorylation of Smad2 to inhibit fibroblast migration (88)
**Type I collagen genes**	The opposite effect of TGF-β and TNF-α on type I collagen gene expression may be related to MAPK (89–92)
**Nucleus pulposus cells**	Reversal of TNF-α-induced increase of MMP3 in NP by TGF-β1 may be associated with ERK1/2 activation (93)
**NP cells**	TGF-β1 antagonizes TNF-α-mediated syndecan-4 upregulation, which is attenuated by inhibitors of ERK1/2 and NF-κB (94)

For fibrosis, both of them play a synergistic role in different tissues. In rat kidney and lung tissues, TNF-α and TGF-β cooperatively induce fibrosis through their respective pathways (74–76), which is related to the expression of Plasminogen activator inhibitor-1 (PAI-1) (95). In the retina, TNF-α activates TGF-β signal transduction to induce the formation of fibrotic lesions in retinal pigment epithelium cells (77), which is related to the promotion of the expression of CD44 and MMP9 (96).

In EMT, TNF-α and TGF-β can stimulate intercellular transformation of bronchial epithelial cells and promote cell migration in combination (78). In addition, TNF-α can also enhance the endothelial mesenchymal transition induced by TGF-β (97, 98). Most of the literature focuses on the synergistic promotion of EMT in tumor cells. In terms of breast cancer, TGF-β and TNF-α can not only induce EMT, but also form a stable breast cancer stem cell phenotype (79, 80). EMT of breast cancer cells is regulated by TGF-β/Smad-dependent pathway and activated by TNF-α/NF-κB/Twist, which both promote tumor metastasis and synergistically promote breast cancer cell migration (99, 100). Activation of gene transcription programs associated with poor prognosis and increased malignancy in breast cancer is associated with activation of the NF-κB signaling pathway (101, 102). In cervical cancer and ovarian cancer, TNF-α and TGF-β induce EMT and cancer stem cell-like properties through the NF-κB/Twist axis (81, 82). In lung cancer, TNF-α enhances TGF-β1-induced EMT and enhances TGF-β1-induced cell contraction (103). In A549 cells, TGF-β1 induces EMT in a Smad-dependent manner, while TNF-α accelerates this process and may be involved in the regulation of miR-23a (83). Combined use of TNF-α and TGF-β can promote the dryness of H460 lung cancer through NF-κB and FoxM1 pathways (84). Moreover, studies have shown that the release of TNF-α and TGF-β1 from macrophages can synergistically increase the migration rate and persistence of 3D cancer cells. TGF-β1 mainly acts *via* the MT1-MMP pathway, while TNF-α acts mainly through the NFκB-MMP1 pathway (85). Therefore, TNF-α and TGF-β play a mutually promoting role in the regulation of fibrosis and EMT, and they complement each other and are two essential factors in the process of tumor formation.

TNF-α and TGF-β not only have synergistic effects on EMT and fibrosis, but also have antagonistic effects in some microenvironments. This antagonism is mainly reflected in fibroblasts, the regulation of type I collagen, nucleus pulposus cells and liver cancer. TGF-β can stimulate the production of extracellular matrix of fibroblasts, while TNF-α has anti-fibrotic activity and TNF-α can down-regulate the level of TGF-βRII protein through proteolysis in human skin fibroblasts, thereby attenuating the effect of extracellular matrix response to TGF-β (86). TNF-α inhibits phosphorylation of Smad2/3 by TGF-β1, thereby inhibiting the differentiation of myofibroblasts (87). In addition, high levels of TNF-α may inhibit fibroblast migration by elevating the level of Smad7 and reducing the phosphorylation of Smad2 (88). TGF-β can enhance the expression of type I collagen gene, while TNF-α has an antagonistic effect on the expression of type I collagen gene, which may be related to the bidirectional regulation of MAPK pathway (89–92). TGF-β1 can reverse the TNF-α-induced increase of MMP3 in nucleus pulposus cells (NP), which may be due to the activation of ERK1/2 signaling pathway, and treatment with ERK1/2 inhibitors (PD98059 and U0126) can abolish the antagonistic effect of TGF-β1 on TNF-α-mediated catabolic response (93). TGF-β1 antagonizes TNF-α -mediated up-regulation of syndecan-4 in NP cells. Treatment with ERK1/2 and NF-κB inhibitors can reduce TNF-α up-regulation of syndecan-4. This suggests that TGF-β1 exerts an anabolic effect on intervertebral discs by inhibiting syndecan-4 expression (94). In terms of liver cancer, TGF-β treatment can induce Huh7 in liver cancer cells to up-regulate autophagy gene expression, strongly activate autophagy and induce EMT, while TNF-α inhibits TGF-β-induced EMT levels by inhibiting autophagy (104).

In kidney, lung tissue and retinal pigment epithelial cells, the two together induce fibrosis; in breast cancer, ovarian cancer, cervical cancer, lung cancer and bronchial epithelial cells, the two together induce EMT. In fibroblasts, TGF-β promotes fibrosis, while TNF-α is anti-fibrotic; in liver cancer Huh7 cells, TGF-β induces EMT, while TNF-α resists EMT.

### Role of TNF-α and TGF-β in Genomic Instability and Mutation

Mitotic abnormalities in normal cells frequently lead to missegregation of chromosomes, which produces genomic instability that triggers the development and progression of tumors (105). TNF-α and TGF-β have dual roles in causing genomic instability and mutations, as shown in **Table 5**. Studies have showed that there is a positive correlation between TNF-α and TGF-β and TP53 mutations (106, 109). Aberrant expression of androgen receptor (AR) -dependent transcriptional programs is a decisive pathology in the development of prostate cancer, and studies have shown that TNF-α and TGF-β can be involved in mediating AR-dependent gene transcription (107). Radiation can induce persistent genomic instability of bone marrow cells in mice, which is associated with high expression of TNF-α with TGF-β (110).TGF-β can up-regulate the activity of the pseudoxanthoma elasticum genesis gene ABCC6 promoter in HepG2 cells, while TNF-α has the opposite effect (108).

**Table 5:** The mechanism of TNF-α and TGF-β on genomic instability and mutations in different cells.

Tissue/cell	Mechanism
**Synergy**	
**Monocyte macrophages**	The two are positively associated with the presence of TP53 mutations (106)
**Androgen Receptor**	The two regulate AR-dependent gene transcription to affect the development of prostate cancer (107)
**HepG2**	TNF-α can inhibit TGF-β from up regulating the activity of ABCC6 promoter (108)

In summary, in monocytes and macrophages and cholangiocarcinoma, the two are positively correlated with TP53 mutant gene; in fibroblasts, the two jointly inhibit gene mutation. In addition, they can also affect the development of prostate cancer by regulating AR-dependent gene transcription. In the treatment of malaria parasites and liver cancer, the two are antagonistic.

### Effect of TNF-α and TGF-β in Other Aspects

In other respects, TNF-α and TGF-β also interact, as shown in **Table 6**. TGF-β1 can alleviate TNF-α-induced intestinal epithelial barrier disorders, and TGF-β1 reduction significantly alleviated TNF-α-induced changes in proteins ZO-1 and occludin, these results showed that TGF-β1 could protect intestinal integrity after challenge with TNF-α (111). TGF-β1 can inhibit the synthesis of prolactin (PRL) in a dose-dependent manner, while TNF-α can stimulate the synthesis and release of prolactin, and the two cytokines antagonize each other, indicating that their relative concentrations can determine whether PRL synthesis is upregulated or downregulated (112). In terms of osteoclast formation, TGF-β enhances the formation of o TNF-α-induced osteoclasts, which may be reflected that TGF-β can induce the expression of SOCS (113–115). In addition to their different relative roles in different tissues or cells, the two also have different changes in different time periods. For example, in 1-5 months after radiotherapy of A549 cells, TGF-β did not change significantly (slightly more in the first month but not statistically significant), but TNF-α showed a significant trend to increase (116).

**Table 6:** The mechanism of TNF-α and TGF-β on other aspects in different cells.

Tissue/cell	Mechanism
**Intestinal epithelium**	TGF-β1 can reduce the changes of ZO-1 and occludin induced by TNF-α (111)
**PRL**	TGF-β1 inhibits the synthesis of prolactin (PRL),whereas TNF-α does the opposite (112)
**Osteoclasts**	The enhancement of TNF-α-induced osteoclast formation by TGF-β may be related to the induction of SOCS expression by TGF-β (113–115)

## The Application of TNF-α and TGF-β in Tumor Treatment

Some progress has been made in animal tumor model experiments. For example, *in vivo* studies in mice and rats demonstrated that low-dose tail vein injection of TNF-α enhanced the anti-tumor activity of pegylated liposomal doxorubicin (117). There are also experiments to enhance the activity of local TNF-α by removing soluble TNF-α receptors *in vitro*, thus taking advantage of the strong antitumor activity of TNF-α (118). Clinical studies have proved that the hemorrhagic necrosis of the tumor can be quickly observed after the combined use of TNF-α and melphalan (119). The results of clinical trials have shown that the use of the TNF-αantagonist etanercept can alleviate the condition of some patients with ovarian cancer (120), but it has not achieved clinical remission in patients with advanced breast cancer (121).

In related experiments with TGF-β, different inhibitors have achieved satisfactory results in cell culture and animal models. TGF-β blocking peptide P114 can significantly reduce tumor growth (122). However, in clinical trials of cancer, the results are poor or inconsistent with animal experiments, which may be due to the differences between tumor-bearing tissues and naturally formed tumor tissues. However, there are few clinical reports on the combination of the two in the treatment of diseases, and it is unknown whether the combination of the two can achieve better efficacy. The following are some drugs we can currently learn about TNF-α and TGF-β. The FDA-approved inhibitors of TNF-α are infliximab, adalimumab, etanercept, golimumab and certolizumab. The role of TGF-β in tumors is complicated, so many inhibitors are in clinical trials. Inhibitors of TGF-β are mainly classified into four categories, TGF-β receptor kinase small molecule inhibitors, monoclonal antibodies that prevent TGF-β binding to receptor complexes, ligand traps, and TGF-β activation inhibitors (123). Several commonly used drugs related to TNF and TGF are shown in **Table 7**.

**Table 7:** Drugs related to TGF-β and TNF-α.

Drugs	Mechanism
**TGF related drugs**	
**LY3022859 (** 124 **)**	Inhibition of TGF-β receptor-mediated activation of transforming growth factor-β signaling.
**Fresolimumab (** 125 **)**	Interfering With Ligand-Receptor Interactions.
**264RAD (** 126 **)**	Blocks integrin-mediated TGF-β activation to target the TGF-β signaling pathway.
**TNF-related drugs**	
**Infliximab,** **Adalimumab,** **Golimumab**	Inhibits binding of TNF to its receptors to neutralize activation of TNFR1 and TNFR2.
**Etanercept**	Prevents TNF binding to TNFR2.
**Certolizumab pegol**	Selectively neutralize TNF-α.

Drugs targeting TNF-α or TGF-β have limitations and side effects. For example, TNF-α inhibitors can cause opportunistic infections, invasive fungal infections, autoimmune diseases and the development of lymphoma, and increase skin derived incidence of solid tumors (127). The side effects of TGF-β are minor, but long-term systemic inhibition of TGF-β therapy can affect wound healing, tissue repair, and anti-inflammatory effects (128). At present, there have been experiments to develop drugs from the perspective of ligand receptors, hoping to improve clinical efficacy. However, TNF-α and TGF-β are in the upstream position of the cellular pathway, and simple blocking of both will affect many downstream protein abnormalities, so it is necessary to specifically block downstream target proteins that cause a pathological feature may be better.

Traditional Chinese medicine (TCM) is a multi-target in the treatment of diseases, and each target can synergize with each other with few side effect, which provides many possibilities for the combination of traditional Chinese and western medicine. The therapeutic effects of Chinese herbs on TNF-α and TGF-β have been reported. Guizhi Fuling capsule can down-regulate the expression of proinflammatory cytokines IL-1β and TNF-α (129). Dachaihu Decoction can reduce TNF-α and TGF-β in nonalcoholic fatty liver model rat (130). Psoralen, can reduce the levels of TGF-β and TNF-α in bleomycin-induced pulmonary fibrosis (131), and it can also inhibit TNF-α-induced inflammation of synovial cells by down-regulating the synthesis of IL-1β protein (132).

According to different purposes, different intervention measures can be taken for TGF-β and TNF-α. For example, in the treatment of tumors, if the two play a synergistic role in promoting tissue or cell proliferation, inflammation, EMT and inhibiting apoptosis, they can be blocked at the same time. However, if the two play a role in inhibiting tissue or cell proliferation, inflammation, EMT and promoting apoptosis, the expression of both can be promoted. Similarly, when two cytokines exert an antagonistic effect, they need to be blocked or promoted according to their current effects on the tumor. At different time points of tumor development, drug intervention can be given according to the changes of the two at different time points. For instance, as mentioned above, it can promote the production of TGF-β in the early stage to achieve the purpose of anti-tumor, but in the middle and late stages of the tumor, it is necessary to inhibit TGF-β to resist tumor angiogenesis. TNF-α also seems to have this logic, that is, it can regulate tumor apoptosis in the early stage, and it can increase tumor invasion and metastasis in the late stage. In most reports, it is described that the two should be used to effectively kill cancer in the early stage (133, 134), and the two should be targeted for treatment in the late stage (135, 136). However, we have proposed that the two play more antagonistic effects in the early stage and evolution of the tumor, and synergistic effects occur in the late stage of the tumor, and whether targeted therapy is needed or not needs to be distinguished according to different tissues and cells.

**Figure 4:**
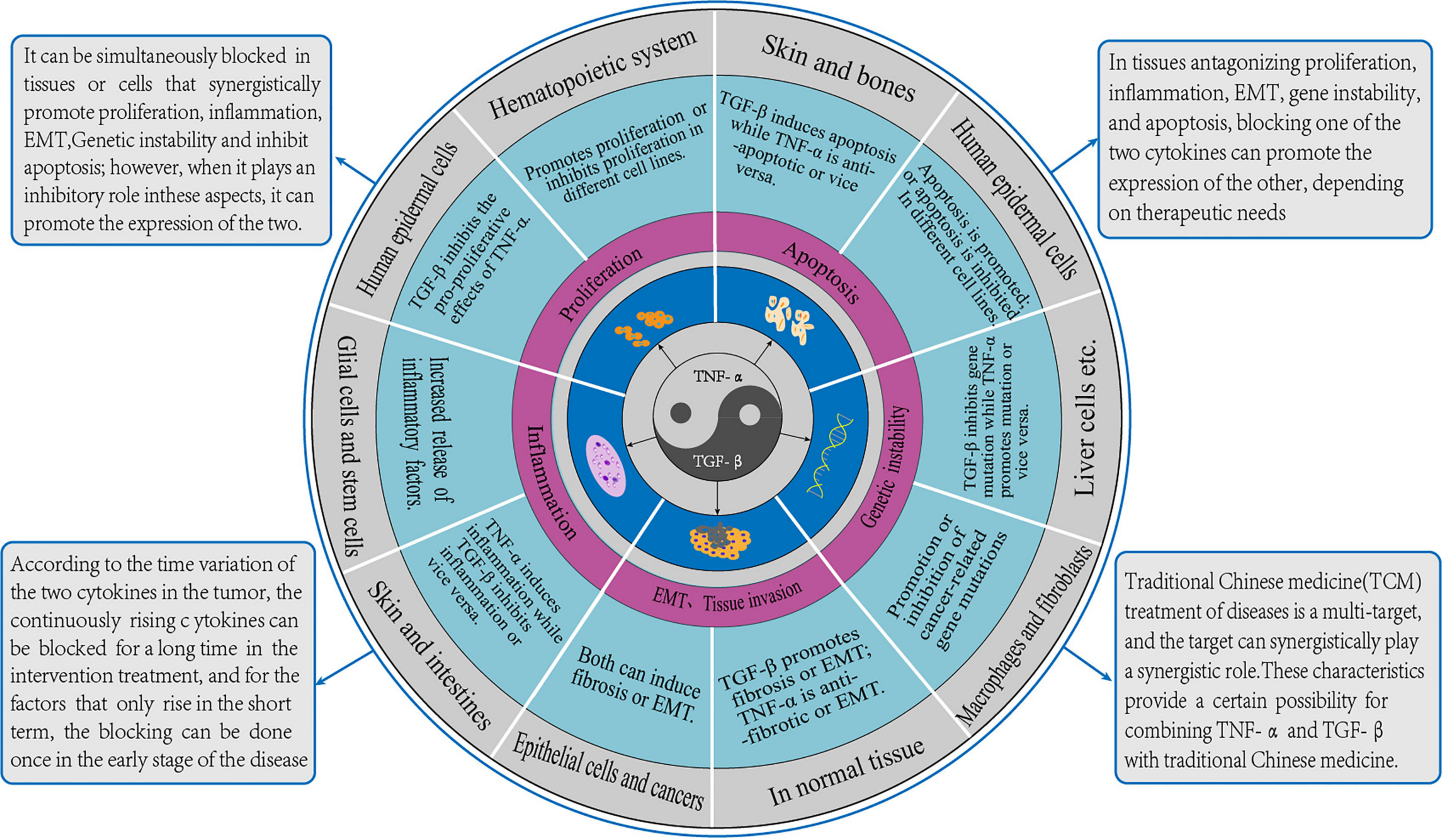
TGF-β and TNF-α action diagram. The crosstalk effect of TGF-β and TNF-α is similar to the relationship between Yin and Yang in Traditional Chinese medicine, which restricts and balances each other. This figure summarizes the roles of the two and their corresponding cell types described in the paper. The methods of synergism, antagonism and time-varying were put forward, and the prospects of integrated traditional Chinese and western medicine were also discussed.

According to the above five effects of TGF-β and TNF-α on tumor genesis and development, we made the following generalizations. In proliferation, the antagonistic effect of the two was mainly in epidermis, and the synergistic effect was mainly in hematopoietic system. In terms of apoptosis, the antagonistic effect of the two was mainly in skin and bone, and the synergistic effect was mainly in glial cells. In terms of inflammatory response, the antagonistic effect is mostly skin and intestinal tract, and the synergistic effect is mostly in glial cells and stem cells. In EMT and fibrosis, the antagonistic effect is mostly in normal tissues, while the synergistic effect is mostly in epithelium and cancer. For genomic instability, the antagonistic effect of the two was mainly in hepatocytes, while the synergistic effect was mainly in fibroblasts and macrophages. So we guess that the co-action of the two is mostly in the endoderm, while the antagonistic action is mostly in the ectoderm. This corresponds to the theory of Yin and Yang and the theory of exterior and interior in Traditional Chinese medicine, as concluded in **Figure 4**.

## Conclusion

In summary, based on the bifacial nature of TNF-α and TGF-β in the body microenvironment, this article describes the interaction between the two in the process of tumor formation from five aspects: proliferation, apoptosis, inflammation, EMT and genomic instability. Summarizing the different functional roles of the two in different microenvironments helps to speculate and control the evolution of cancer. For decades, researchers have been committed to the study of cytokines to treat cancer-related diseases. When designing relevant treatment methods, the direct effects of cytokines and the mutual regulation of multiple factors in immunity must be considered. In addition, traditional Chinese medicine has the advantages of multiple points of marked effect, synergy, and less side effects, which can supplement the inadequate treatment of modern medicine, and it also provides many possibilities for the combined application of traditional Chinese medicine with TNF-α and TGF-β.

## Author Contributions

Z-wL and Y-mZ are responsible for the literature review and article writing. L-yZ and TZ are responsible for the guidance and article modification. Y-yL, G-cZ, Z-mM, and MS are responsible for the collection and sorting of materials. J-pH and ND are responsible for the guidance of ideas. Y-qL provide thought guidance and drafts. All authors contributed to the article and approved the submitted version.

## Funding

National Natural Science Foundation of China (No. 81973595); National Natural Science Foundation of China (No. 82004094); Support Program for Longyuan Youth and Fundamental Research Funds for the Universities of Gansu Province. (No. GZTZ[2021]17-1); Provincial university industry support project in Gansu (No. 2020C-15).

## Conflict of Interest

The authors declare that the research was conducted in the absence of any commercial or financial relationships that could be construed as a potential conflict of interest.

## Publisher’s Note

All claims expressed in this article are solely those of the authors and do not necessarily represent those of their affiliated organizations, or those of the publisher, the editors and the reviewers. Any product that may be evaluated in this article, or claim that may be made by its manufacturer, is not guaranteed or endorsed by the publisher.
